# Silver nano-reporter enables simple and ultrasensitive profiling of microRNAs on a nanoflower-like microelectrode array on glass

**DOI:** 10.1186/s12951-022-01664-7

**Published:** 2022-10-23

**Authors:** Ying Gan, Mingxing Zhou, Huiqiang Ma, Jiameng Gong, Shan-Yu Fung, Xian Huang, Hong Yang

**Affiliations:** 1 The Province and Ministry Co-Sponsored Collaborative Innovation Center for Medical Epigenetics, Department of Pharmacology, School of Basic Medical Sciences, School of Biomedical Engineering, Intensive Care Unit, The Second Hospital, Tianjin Medical University, No. 22 Qixiangtai Road, Heping District, Tianjin, 300070 China; 2 Department of Biomedical Engineering, Tianjin University, 92 Weijin Road, Tianjin, 300072 China; 3 Department of Immunology, School of Basic Medical Sciences, Tianjin Medical University, No. 22 Qixiangtai Road, Heping District, Tianjin, 300070 China

**Keywords:** Electrochemical biosensor, Microarray, Nanostructured electrodes, microRNAs, Silver nanoparticle

## Abstract

MicroRNAs (miRNAs) are small non-coding RNAs with ~ 22 nucleotides, playing important roles in the post-transcriptional regulation of gene expression. The expression profiles of many miRNAs are closely related to the occurrence and progression of cancer and can be used as biomarkers for cancer diagnosis and prognosis. However, their intrinsic properties, such as short length, low abundance and high sequence homology, represent great challenges in miRNA detection of clinical samples. To overcome these challenges, we developed a simple, ultrasensitive detection platform of electrochemical miRNAs chip (e-miRchip) with a novel signal amplification strategy using silver nanoparticle reporters (AgNRs) for multiplexed, direct, electronic profiling of miRNAs. A two-step hybridization strategy was used to detect miRNAs, where the target miRNA hybridizes with a stem-loop probe to unlock the probe first, and the opened stem-loop can further hybridize with AgNRs for signaling amplification. To enhance the detection sensitivity, the gold nanoflower electrodes (GNEs) were constructed in the microaperture arrays of the e-miRchips by electroplating. With the optimal size of the GNEs, the e-miRchip showed excellent performance for miR-21 detection with a detection limit of 0.56 fM and a linear range extended from 1 fM to 10 pM. The e-miRchip also exhibited good specificity in differentiating the 3-base mismatched sequences of the target miRNA. In addition, the e-miRchip was able to directly detect miR-21 expression in the total RNA extracts or cell lysates collected from lung cancer cells and normal cells. This work demonstrated the developed e-miRchip as an efficient and promising miniaturized point-of-care diagnostic device for the early diagnosis and prognosis of cancers.

**Graphical Abstract:**

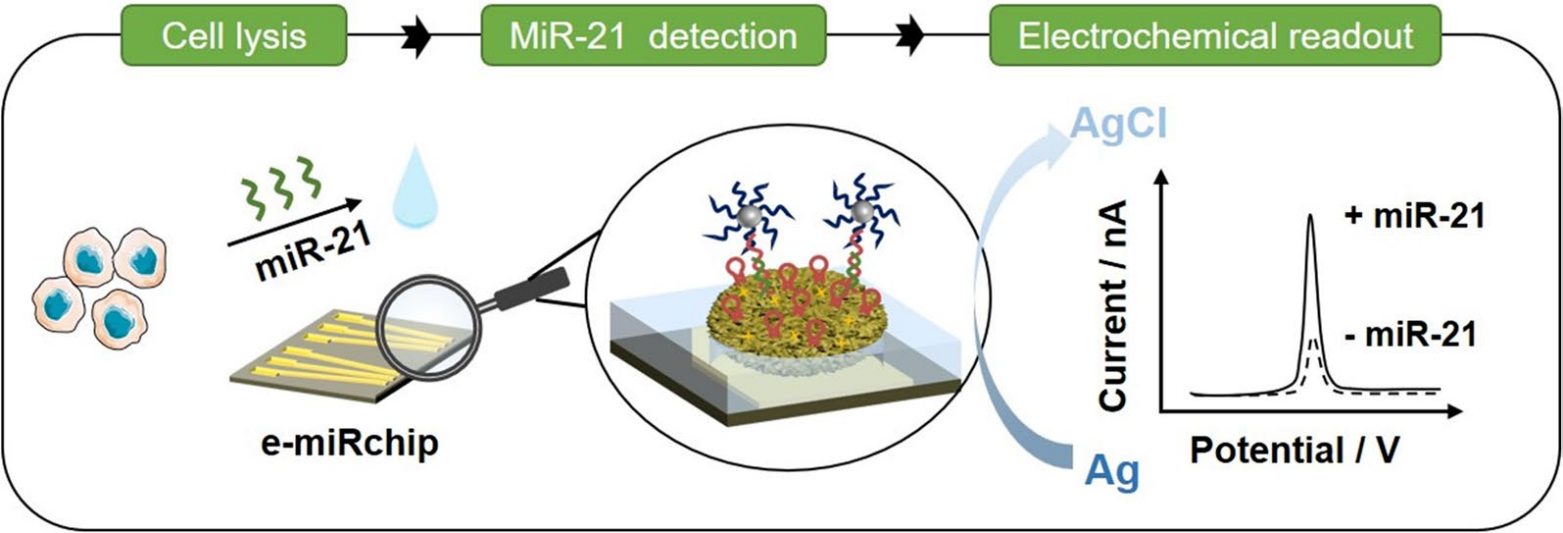

**Supplementary Information:**

The online version contains supplementary material available at 10.1186/s12951-022-01664-7.

## Introduction

Early diagnosis and precise prognosis of cancer have become one key factor in order to increase the success rate in cancer treatment [1]. A variety of biological components in the blood or body fluids, such as circulating tumor cells, microRNAs (miRNAs) and DNA, have been identified as tumor biomarkers for early diagnosis of cancer [2, 3]. Among these biomarkers, miRNAs, a class of small non-coding RNAs consisting of approximately 22 nucleotides [4], have shown great promise for monitoring the status of tumor development, as the dysregulation of certain miRNAs expression is often associated with the onset of cancer [5, 6]. However, the intrinsic properties of miRNAs, such as the short length, low abundance, and high sequence homology, represent great challenges in analyzing miRNAs from clinical samples.

Although the reverse transcription-quantitative polymerase chain reaction (RT-qPCR) is the well-known gold standard method for miRNA analysis, it is not suitable for rapid and multiplexed miRNA detection for fast clinical diagnosis and prognosis. This is because the sample processing for RT-qPCR (including RNA extraction and purification, target molecule elongation or stem-loop primer design, reverse transcription, and enzyme amplification) is time-consuming and not cost-effective [5, 7]. Thus, many nanotechnology-based gene sensing methods coupled with electrochemical readouts are emerging to hopefully overcome these problems. These electrochemical devices can achieve cost-effective, sensitive, rapid and direct quantitative analysis of genetic samples, promoting the rapid development of point-of-care testing (POCT) techniques for clinical uses [8].

With the advances in nano-fabrication, various miniaturized molecular sensing platforms have been fabricated for electrochemical gene detection [9, 10]. These nanostructured devices are known for performing high throughput measurements, and having small resistor–capacitor constants and high mass-transfer rate [11]. For example, Li’s group designed a single nanowire electrode using dual-signal amplification strategy of methylene blue [6] and ferrocene (Fc) for miRNA-16 detection with a low detection limit of 16 fM [12]. Hou’s group fabricated a DNA-circuit strip based on a carbon paper electrode made of graphene and gold nanoparticles to detect as low as 21.4 aM of miRNA-21 under the help of duplex-specific nuclease [13]. In addition, Kelley’s group [14–17] and Soleymani’s group [18, 19] proposed a multiplexed detection platform with gold nanostructured electrodes that significantly improved the sensitivity of gene detection to attomolar range. These works set good examples of nanostructured electrochemical devices for robust gene and miRNA detection.

In the attempts to enhance the performance of molecular sensing, metal nanoparticles are often used for their unique chemical and physical properties [20, 21]. Silver nanoparticles (AgNPs) are commonly employed in electrochemical sensors because they can participate in the solid-state Ag/AgCl redox reaction in the presence of chloride to generate strong redox signals [22, 23]. To further boost the sensitivity of molecular sensing, some amplification strategies have been applied for the AgNPs-based gene sensors, including the rolling circle amplification (RCA) [24], hybridization chain reaction (HCR) [25, 26], and duplex-specific nuclease (DSN)-based and strand displacement reaction (SDR)-based and tetrahedral DNA nanostructure-based amplification processes [27, 28]; however, these strategies may increase the detection time and complicate the operation procedure, impeding their fast clinical uses.

Herein, we aimed to develop an ultrasensitive, multiplexed, facile-to-fabricate and easy-to-operate electrochemical miRNA profiling chip (e-miRchip). The device was designed with three unique features: (1) a patterned gold array with microapertures on a glass slide; (2) gold nanoflower electrodes (GNEs) were plated inside the microapertures serving as robust sensing elements; and (3) AgNP-based reporters (AgNRs) were integrated for signal reporting and amplification. We first fabricated the e-miRchip with patterned gold on the glass slide using the advanced photolithography technology. The nanostructured electrodes and AgNR system were then integrated to the chip, and the system was optimized for the detection of the cancer biomarker miRNA-21 (miR-21). With the optimized conditions, the e-miRchip exhibited excellent detection performance with a detection limit of 0.56 fM and a wide dynamic range. Furthermore, it was able to differentiate the mismatched sequence or random sequence from the fully complementary one. More importantly, the e-miRchip was capable of analyzing miR-21 levels in complexed biological samples. This work demonstrated a simple, miniatured and multiplexed electrochemical device integrated with AgNP-mediated amplification strategy for direct and ultrasensitive miRNAs detection in tumor cell lysates. We believe that the e-miRchip platform represents a highly efficient and promising point-of-care diagnostic device for the early diagnosis of cancer.

## Experiments and methods

### Chemicals and reagents

All reagents used in this work were analytical grade unless otherwise noted. Chloroauric acid (HAuCl_4_·3H_2_O), hexaammineruthenium (III) chloride (Ru(NH_3_)_6_Cl_3_), potassium chloride (KCl), 6-mercapto-1-hexanol (MCH), were purchased from Sigma-Aldrich (St. Louis, MO, USA). Tris(hydroxymethyl)aminomethane, sodium chloride (NaCl), magnesium chloride (MgCl_2_) and nucleic acids were from Sangon Biotech (Shanghai, China). Silver nitrate (AgNO_3_) was bought from Yingda Rare Chemical Reagents Factory (Tianjin, China). Sodium citrate tribasic dihydrate (C_6_H_5_O_7_Na_3_·2H_2_O) and tannic acid (C_76_H_52_O_46_) were purchased from BBI (Shanghai, China). Diethyl pyrocarbonate (DEPC) was obtained from Solarbio (Beijing, China). Dulbecco's modified eagle medium (DMEM) was purchased from XP Biomed (Shanghai, China). Fetal bovine serum (FBS) was obtained from Gibco (Grand Island, NY, USA). Total RNA was extracted by RNeasy Mini kit obtained from Qiagen (Hilden, Germany). Ultrapure water was produced by Millipore Milli-Q water purification system. RT-qPCR was performed using Taqman™ Universal PCR Master Mix II and MicroRNA Reverse Transcription Kit (Thermo Scientific, Waltham, MA, USA). MiRNA levels were measured with Taqman™ MicroRNA Assays (Thermo Scientific, Waltham, MA, USA). The miR-21 (Assay ID: 000397) and snRNA-U6 (Assay ID: 001973) serve as the target gene and the housekeeping gene, respectively.

The sequences (5′–3′) of the synthesized DNA and RNA are listed in Table 1.

**Table 1:** The sequences of the synthesized DNA and RNA

DNA/RNA	Sequence
SLP (stem-loop probe)	HS-C6-GGCCGTCAACATCAGTCTGATAAGCTAAACATGATGACGGCC
DNA-21	TAGCTTATCAGACTGATGTTGA
MiR-21	UAGCUUAUCAGACUGAUGUUGA
Signal probe	HS-C6-TTTTTGGCCGTCA
Mismatched sequence	TAGCTTATCAGACTGATGTGAC
Random 1	AATACCCCACCACCTTTTGA
Random 2	GCAAACGAGACATCATAGGCA

### Synthesis of AgNRs

The AgNRs were prepared by modifying AgNPs with signal probes on the surface. First, bare AgNPs were synthesized based on a modified method in the literature [29]. Briefly, mixture (50 mL) of trisodium citrate (5 mM) and tannic acid (0.01 mM) solution was boiled under stirring (700 rpm). Then, AgNO_3_ solution (500 μL, 25 mM) was added to the mixture, and the solution turned into yellow color overtime. After 15 min, the solution was cooled to room temperature and stored at 4 °C. Next, the bare AgNPs were mixed with the signal probes and incubated for 48 h in the presence of 0.1 M NaCl. The probe modified AgNPs (AgNRs) were washed with pure water and centrifuged at 12,000 rpm for 10 min twice. The collected AgNRs were redispersed in pure water and stored at 4 °C prior to use.

### Physicochemical characterization of AgNPs and AgNRs

The morphology and size of AgNPs and AgNRs were characterized by transmission electron microscope (TEM, JEOL, Tokyo, Japan) with an accelerating voltage of 80 kV, and dynamic light scattering (DLS) analysis (Zetasizer, Malvern Instruments, Worcestershire, UK); their characteristic absorption peak at ~ 406 nm was measured by the UV–vis spectrophotometer (HITACHI, Tokyo, Japan). The concentration of signal probes was measured by Nanodrop Lite Spectrophotometer (Thermo Scientific, Waltham, MA, USA).

### Fabrication of the electronic chip with microaperture array

Microfabrication technology was used to construct the electronic chip featured with microaperture arrays. The fabrication process began with the deposition of a Cr/Au (5 nm/200 nm in thickness) metal layer through sputtering on a glass substrate. The AZ4620 photoresist was then spin-coated and patterned by lithography for subsequent etching. Patterns of the sensing area were made through wet etching of Cr/Au metal layer. Finally, the parylene insulating layer (1 μm) was deposited on the whole arrays followed by photolithography and reactive ion etching [30] to expose every aperture electrode with a diameter of 10 μm. The aperture was characterized by a scanning electron microscope (SEM, Carl Zeiss, Oberkochen, Germany) with electron high tension (EHT) of 10 kV.

### Electrochemical measurements

The electrochemical signal was measured by Chi660e electrochemical workstation (Chen Hua Instrument, Shanghai, China) with a three-electrode system composed of an Ag/AgCl reference electrode. The cyclic voltammetry (CV) for bare apertures, newly plated GNEs and SLPs functionalized GNEs on the electronic chip was collected in the solution containing 2 mM K_3_[Fe(CN)_6_] and 0.1 M KCl with the potential range from − 0.1 to 0.5 V and the scan rate of 0.05 V/s. The electrochemical impedance spectroscopy (EIS) was conducted in 2 mM K_3_[Fe(CN)_6_]/K_4_[Fe(CN)_6_] and 0.1 M KCl solution at the bias potential of 0.208 V, the amplitude of applied voltage of 10 mV, and the frequency from 0.1 Hz to 10^6^ Hz. The CV for collecting the oxidation and reduction currents of the hybridized AgNRs on the GNEs was performed in 0.3 M KCl with the potential range from − 0.1 to 0.7 V and the scan rate of 0.05 V/s.

The electrochemical active surface area (ECSA) was calculated from the reduction peak of GNEs according to the following equation [31]:

1 $$ECSA={\int }_{PEAK}I dV/C,$$

where ${\int }_{PEAK}I dV$ is the integration of reduction peak, C is a conversion factor of 482 μCcm^−2^.

The SLP surface density was calculated using chronocoulometry in 1 mM PBS in the presence and absence of 50 μM [Ru(NH_3_)_6_]^3+^ with a pulse width of 0.25 s and a potential step from 0.2 to − 0.45 V. The SLP surface density was obtained according to the protocol established by Steel and the Cottrell equation [32]:

2 $$Q=\frac{2nFA{{D}_{0}}^{1/2}{C}_{0}^{*}}{{\pi }^{1/2}}{t}^{1/2}+{Q}_{dl}+nFA{\Gamma }_{0},$$

where *n* is the number of electrons per molecule for reduction, *F* is the Faraday constant (C/mole), *A* is the electrode area (cm^2^), *D*_*0*_ is the diffusion coefficient (cm^2^/s), ${C}_{0}^{*}$ is the bulk concentration of [Ru(NH_3_)_6_]^3+^ (mole/cm^3^), *Q*_*dl*_ is the capacitive charge, and *Γ*_*0*_ is the amount of surface-absorbed [Ru(NH_3_)_6_]^3+^ (mole/cm^2^). Chronocoulometric data is plotted as *Q* versus ${t}^{1/2}$. The intercepts of this curve in the absence and presence of [Ru(NH_3_)_6_]^3+^ are ${Q}_{dl}$ and ${Q}_{dl}+nFA{\Gamma }_{0}$, respectively. ${\Gamma }_{0}$ can be calculated from the difference between these two intercepts. The surface density (${\Gamma }_{SLP}$) can be obtained according to the following equation:

3 $${\Gamma }_{SLP}={\Gamma }_{0}\left(\frac{z}{m}\right){N}_{A},$$

where z is the charge of [Ru(NH_3_)_6_]^3+^, m is the number of bases in SLP.

### E-miRchip fabrication and miR-21 detection

The e-miRchip was made by a two-step process: the GNE deposition followed by the surface functionalization. In the first step, the chip was cleaned with acetone, isopropanol and water for 1 min and blown dry completely with N_2_. The GNE deposition was performed with the chronoamperometry in the electrolytes of HAuCl_4_ (50 mM) and HCl (0.5 M) with a deposition potential of 0.5 V. The GNEs grew from the aperture one by one. The size of GNE was controlled by adjusting the deposition time, and the GNE was characterized by SEM. Specially, to obtain the side views of plated GNE, the 3°-tilted SEM imaging was taken.

For GNE surface functionalization, SLPs (5 μM) were prepared with NaCl (1 M) and Tris–HCl (10 mM) solution. The SLPs were heated at 95 ℃ for 10 min and then cooled to room temperature (~ 1 h) to form the stem-loop structure. SLPs (5 μL, 5 μM) were dropped onto the GNE and incubated overnight. MCH (100 μM) was then dropped to block the unbounded sites followed by rinsing with PBS buffer.

MiR-21 was dissolved at various concentrations in the hybridization buffer containing 10 mM PBS, 1 M NaCl and 40 mM MgCl_2_. The e-miRchip was incubated with the miR-21 solution for 1 h at 37 °C. AgNRs (5 μL, 1 nM) were then added and incubated for another 1 h to hybridize with the opened SLPs. Finally, the e-miRchip was thoroughly rinsed with 10 mM PBS before electrochemical measurements.

### Cell culture, RNA isolation and direct cell lysis

A549 cells were cultured in DMEM containing 10% FBS and 1% penicillin/streptomycin. HEK293T cells were cultured in DMEM containing 10% FBS. All the cells were cultured at 37 °C with 5% CO_2_. After cell confluence reached to 70–80%, cells were collected for total RNA extraction.

The total RNA was extracted by RNeasy plus mini kit and its concentration was determined by Nanodrop Lite Spectrophotometer (Thermo Scientific, Waltham, MA, USA). For direct detection of miR-21 in the cell lysate, the collected cells (1 × 10^7^) were incubated in the lysis buffer (200 μL, 1% Triton-X 100, 10 mM Tris–HCl, DEPC) for 5 min on ice and then centrifuged to remove the cell debris [33, 34].

### Quantification of miR-21 expression in biological samples by e-miRchip and RT-qPCR

For electrochemical detection, the extracted total RNA was diluted with hybridization buffer to the concentrations of 1, 10 and 100 ng/μL and then was dropped on the e-miRchip. For direct detection of miR-21 in the unpurified cell lysate, the cell lysate was first diluted 1000 times with hybridization buffer and the diluted sample (20 μL) was then placed on the e-miRchip for hybridization. The detection procedure and methods are the same as described in the previous section for miR-21 detection.

The expression of miR-21 in the total RNA of A549 and HEK293T cells was also analyzed by RT-qPCR. The extracted total RNA was reversely transcribed to complementary DNA (cDNA) using MicroRNA Reverse Transcription Kit. Quantitative PCR was performed on cDNA using Taqman™ Universal PCR Master Mix II and Taqman™ MicroRNA Assays on a QuanStudio™ Real-Time PCR System (Thermo Scientific, Waltham, MA, USA). The data were automatically analyzed by QuanStudio software. SnRNA-U6 serves as the housekeeping gene [35]. The miR-21 relative expression in A549 and HEK293T cells was calculated based on the cycle threshold (Ct) values as ratio = 2^−ΔΔCt^.

### Statistical analysis

The GraphPad Prism 7.0 was used for statistical analysis. All data were expressed as means ± SEM, and P < 0.05 was considered statistically significant. For two groups comparison, the student t-test was performed, while the one-way ANOVA with Bonferroni post-test was used for multiple comparisons. Nyquist plots of EIS were fitted and analyzed by Zview 3.0 according to the Randle’s equivalent circuit.

## Results and discussion

### Design and fabrication of e-miRchip

**Scheme 1:**
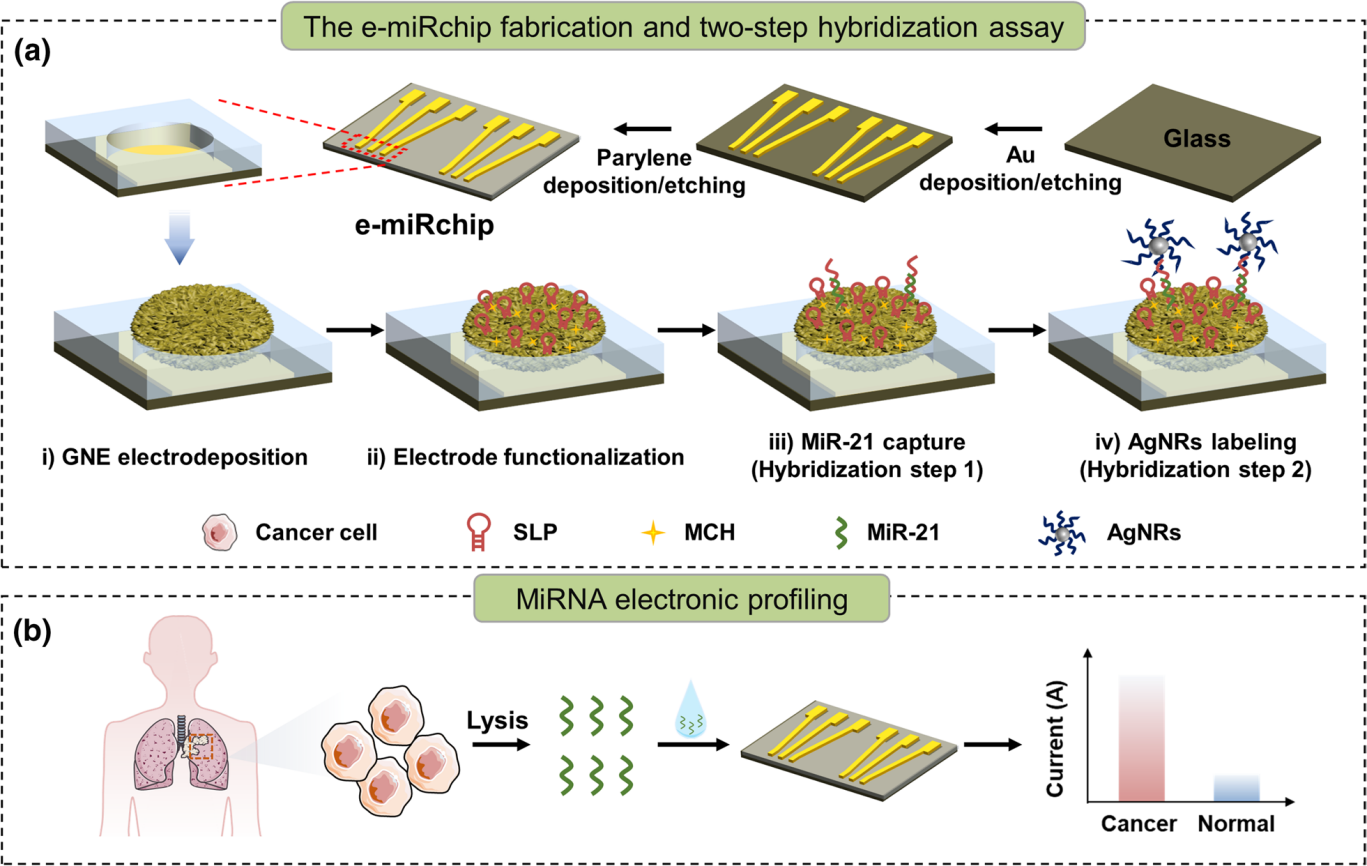
Proposed e-miRchip platform for miRNA analysis. **a** The e-miRchip was fabricated with photolithography with the deposition and etching of the conductive gold and insulating parylene layers on glass to make the designed microaperture array. Electrodeposition of gold was performed to fabricate GNEs in the microaperture (i), and the GNEs were functionalized with SLP immobilization and 6-mercapto-1-hexanol (MCH) backfilling (ii). The miR-21 was captured by the SLPs (iii) and the signals were amplified with the AgNRs (iv). **b** Direct, ultrasensitive detection of miR-21 expression in lung cancer. The expression of miR-21 from the lung cancer cells is examined by direct electrochemical measurement on the e-miRchip

We endeavored to develop a new electronic device (e-miRchip) that is facile to fabricate and convenient to operate for direct microRNA profiling in small biological samples with high sensitivity and accuracy. As shown in the Scheme 1, the e-miRchip was designed with gold patterns on top of a glass slide and fabricated by the deposition and etching of Au and parylene layer. The narrow end of the gold pattern has an aperture, in which a gold nanoflower electrode (GNE) grown with electroplating (Scheme 1a). The GNE was then functionalized with the stem-loop probes (SLPs) for the two-step detection of miR-21: the miR-21 first hybridizes with the SLPs to open the loop followed by the second hybridization of opened SLPs with the signal probes on AgNRs for signal amplification. This two-step hybridization enables a one-to-one relationship between miRNA and AgNRs labeling, which can further trigger the amplification cascade based on AgNRs oxidation on GNE for ultrasensitive detection of miR-21. With this sensing strategy, the e-miRchip allows for direct detection of miR-21 in heterogeneous biological samples with high sensitivity and specificity, and could provide a promising method to profile miRNA biomarkers for the early diagnosis and prognosis of cancers (Scheme 1b).

**Fig. 1:**
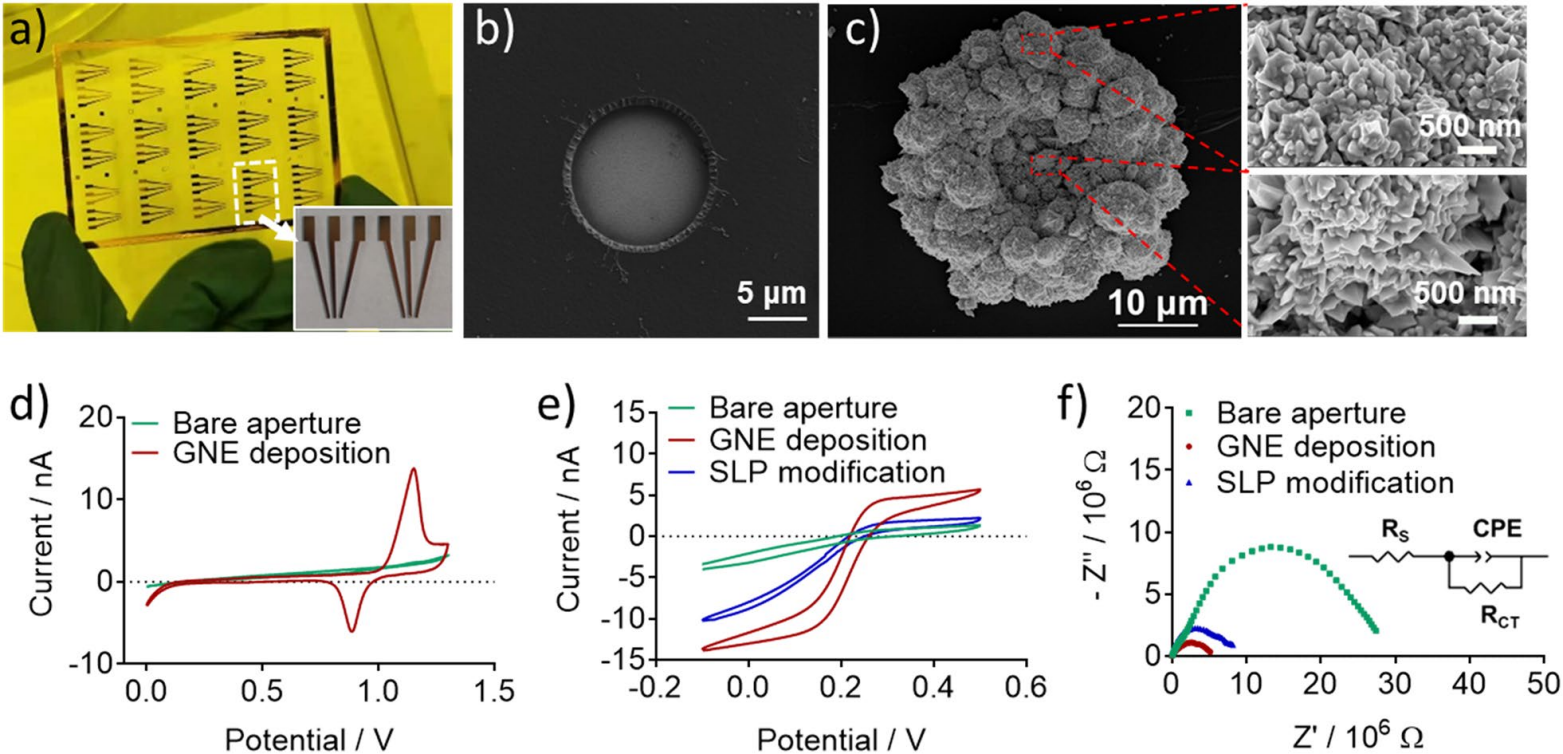
Characterization of e-miRchip and the GNE grown on the microaperture arrays. **a** Photograph of the microaperture array with 15 detection units (Inset: a zoom-in view of the detection unit in the white box labeled area). Each unit contained 6 apertures divided into 2 groups. **b** The SEM image of the bare microaperture. **c** SEM images of the GNE and the zoom-in high-resolution images of the red box labeled regions. **d** CV curves before and after GNE deposition (200 s) in 10 mM H_2_SO_4_ with the scan rate of 0.05 V/s. **e** CV curves of the e-miRchips with the bare aperture, GNE deposition and SLP immobilization on the GNE in 2 mM K_3_[Fe(CN)_6_] and 0.1 M KCl solution with the scan rate of 0.05 V/s. **f** EIS spectra of the e-miRchips in 2 mM K_3_[Fe(CN)_6_]/K_4_[Fe(CN)_6_] and 0.1 M KCl solution. Inset: Randle’s equivalent circuit, Rs: solution resistance, CPE: constant phase angle element, R_CT_: charge-transfer resistance

We adopted a microfabrication approach and prepared the above designed device. With photolithography, the device was made by depositing a pattern of gold layer on top of a glass slide to provide a set of conducting leads and external contacts for multiplexed electronic analysis. The photograph of the fabricated microaperture array was shown in Fig. 1a. The array contained 15 detection units with a size of 1.2 cm × 1 cm labeled with white box. Each unit (Fig. 1a, inset) consisted of 6 apertures for GNEs electrodeposition at the tip of the leads; the apertures were divided into two groups with three replicates in each for testing two different samples. The diameter of the aperture was measured to be about 12 μm by SEM (Fig. 1b) and the insulating layer around the aperture was in good shape without any breakage. To increase the electrochemical active surface area (ECSA) of the e-miRchip for efficient SLP immobilization, GNE was plated in the aperture using a small overpotential of 0.5 V to form a flower-like nanostructured microelectrode with a diameter of about 40 μm (Fig. 1c). It is known that smaller overpotential facilitates the formation of a compact structure while larger overpotential enables the formation of the dendrite structure [36] due to the balance between charge transfer and mass transport [37]. The zoom-in SEM images of the red box labeled area also showed the nanoflake-like features of the GNE (Fig. 1c). A possible reason for this phenomenon was that the charge transfer rate was limited under a small overpotential, leading to a small crystal-growth rate, which resulted in the relative uniform growth of GNE [37]. Compared with the dendrite structure, the compact structure of GNE is expected to be more robust with unique nanostructures on the surface, which could provide consistent and stable sensing signals.

The electrochemical properties of the GNEs on the e-miRchip were characterized. First, the CV scan of the electrode in 10 mM H_2_SO_4_ showed that the typical features of the gold electrode with the oxidation peak at around 1.2 V and the reduction peak at around 0.9 V [38] were only present with GNE deposition (Fig. 1d), confirming the formation of GNE in the aperture with highly increased ECSA. Next, the SLPs immobilized GNE was assessed by CV measurements. For CV scans in 2 mM K_3_[Fe(CN)_6_] solution, S-shape curves were observed as the typical feature for microelectrode [39, 40] (Fig. 1e). The diffusion-limited steady-state current [40] of GNE was about 4 times of the bare aperture, indicating that GNE could generate excellent electrochemical signals. Interestingly, a decreased I_SS_ was observed after SLP immobilization on the GNE. This was because many negative charges were introduced to the GNE surface by SLP immobilization, which repulsed the negatively charged [Fe(CN)_6_]^3−^ to reduce the current flow. To obtain more direct information of the electrochemical properties of the electrode surface after SLP modification, the EIS was applied (Fig. 1f). The charge-transfer resistance (R_CT_) was extracted through Randle’s equivalent circuit, corresponding to the diameter of the semicircular in the spectrum. R_CT_ reflects difficulty of electron transfer from [Fe(CN)_6_]^3−/4−^ to the electrode [41]. Upon GNE deposition, R_CT_ decreased from 26.9 × 10^6^ Ω to 5.38 × 10^6^ Ω, indicating that GNE increased the ECSA and mass transport significantly. However, after SLP modification, R_CT_ increased to 7.93 × 10^6^ Ω, due to the negative charges introduced by SLPs, which hindered the electron transfer and diffusion of [Fe(CN)_6_]^3/4−^ to the GNE. These observations confirmed that the SLPs were successfully immobilized on the GNE.

### The performance of the synthesized AgNRs for miRNA sensing

**Fig. 2:**
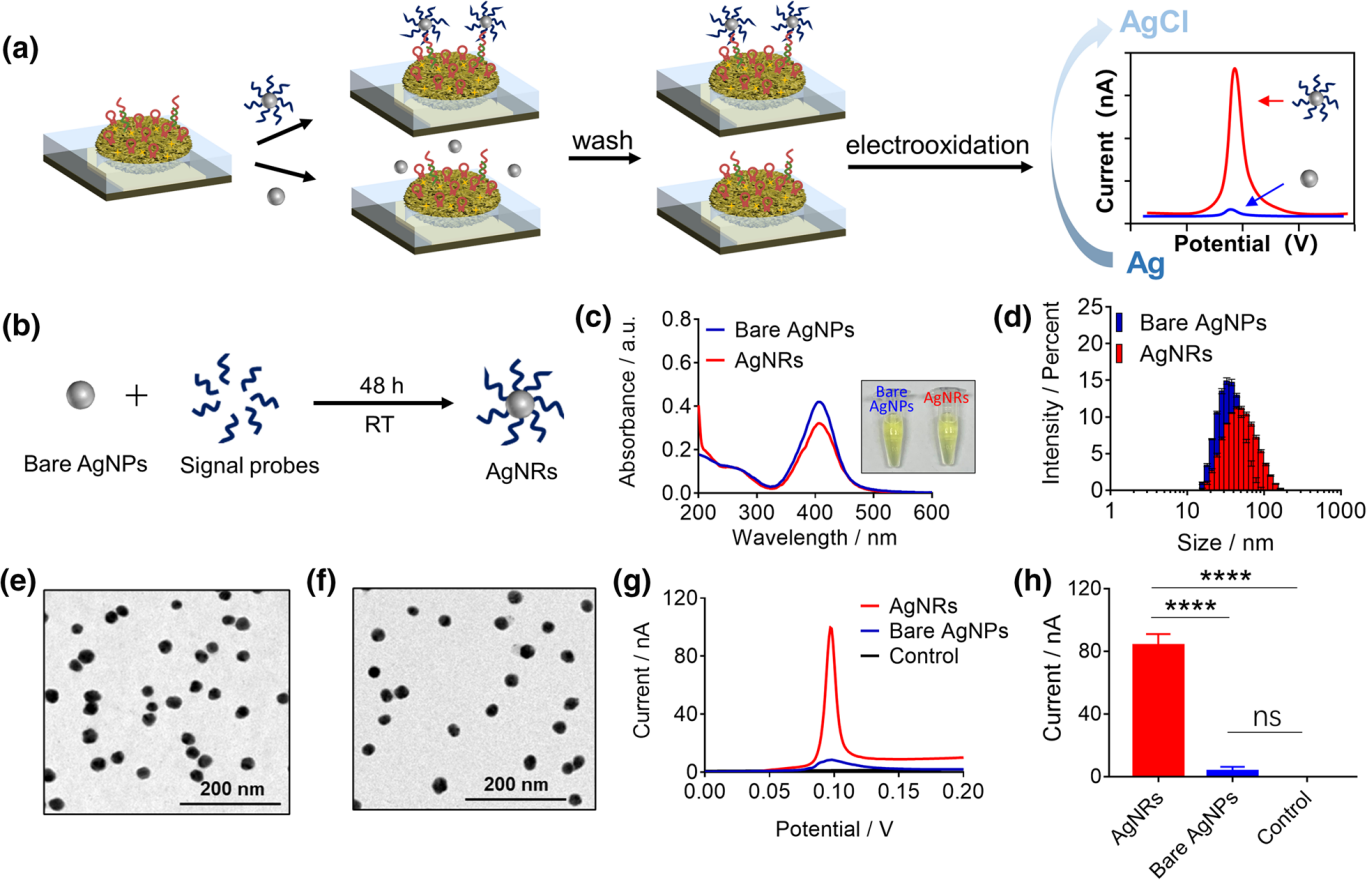
The performance of the synthesized AgNRs for miRNA sensing. **a** A schematic diagram of AgNRs (vs. the bare AgNPs) in reporting and amplifying the signals of the hybridization of miRNAs via the electrooxidation process. **b** A schematic diagram of AgNR synthesis. **c** UV–vis spectra and the representative photographs (inset) of the bare AgNPs and the AgNRs in 10 mM PBS. **d** The hydrodynamic diameter of the bare AgNPs and AgNRs by DLS measurement. The TEM images of the bare AgNPs (**e**) and AgNRs (**f**). **g** CV scans after AgNPs or AgNRs added on the e-miRchip. The control group represents the solvent for nano-reporters (10 mM PBS). **h** The comparison of the oxidation peak current obtained from (**g**). N ≥ 3 per group, ****p < 0.0001

To report and amplify the signals of miRNA hybridization with the immobilized SLPs on the GNE, the AgNRs were utilized in the e-miRchip system. The signal reporting of AgNRs was through the electrooxidation process (Fig. 2a). The AgNRs were fabricated by modifying the surface of the bare AgNPs with the signal probes, capable of hybridizing with the SLPs (Fig. 2b). The photographs of AgNRs in the PBS solution (10 mM) show a clear yellow color before and after signal probes conjugation. The UV–vis absorption spectra of both the bare AgNPs and AgNRs showed a characteristic peak at 406 nm (Fig. 2c) [42]; the presence of the signal probes on AgNRs did not affect the absorption peak position. The observed slight decrease in the peak intensity of AgNRs might be due to the loss of AgNRs during the fabrication process. From the UV–vis absorption intensity and the reported extinction coefficient (4.18 × 10^9^ L mol^−1^ cm^−1^) [43], the concentration of the synthesized AgNPs was calculated to be about 1 nM. Their hydrodynamic size and dispersibility were characterized by DLS as shown in Fig. 2d. Both the unmodified AgNPs and AgNRs had a single characteristic peak with polydispersity index (PDI) of 0.20 ± 0.02 and 0.21 ± 0.02, respectively, indicating uniform dispersibility. Upon the modification with the signal probes, the peak position of AgNRs shifted from 32.80 ± 0.69 nm to 48.40 ± 3.25 nm, confirming the presence of signal probes on the AgNRs. From the TEM image, the size of AgNPs was measured to be 20 nm ± 3.1 nm, and the morphology did not change upon the probe modification (Fig. 2e, f). These results demonstrated that the stable AgNRs were successfully fabricated by conjugating the signal probes onto the synthesized AgNPs. To further verify if AgNRs could report the miRNA hybridization, we compared the CV signals with the addition of AgNRs or bare AgNPs on top of the chips after DNA-21 (the corresponding DNA sequence of miR-21) hybridization. The results showed that the oxidation signals were observed only in the presence of AgNRs, confirming the capability of AgNRs in reporting the miRNAs hybridization (Fig. 2g, h).

### The optimization of the GNE fabrication for nucleic acid sensing

**Fig.3:**
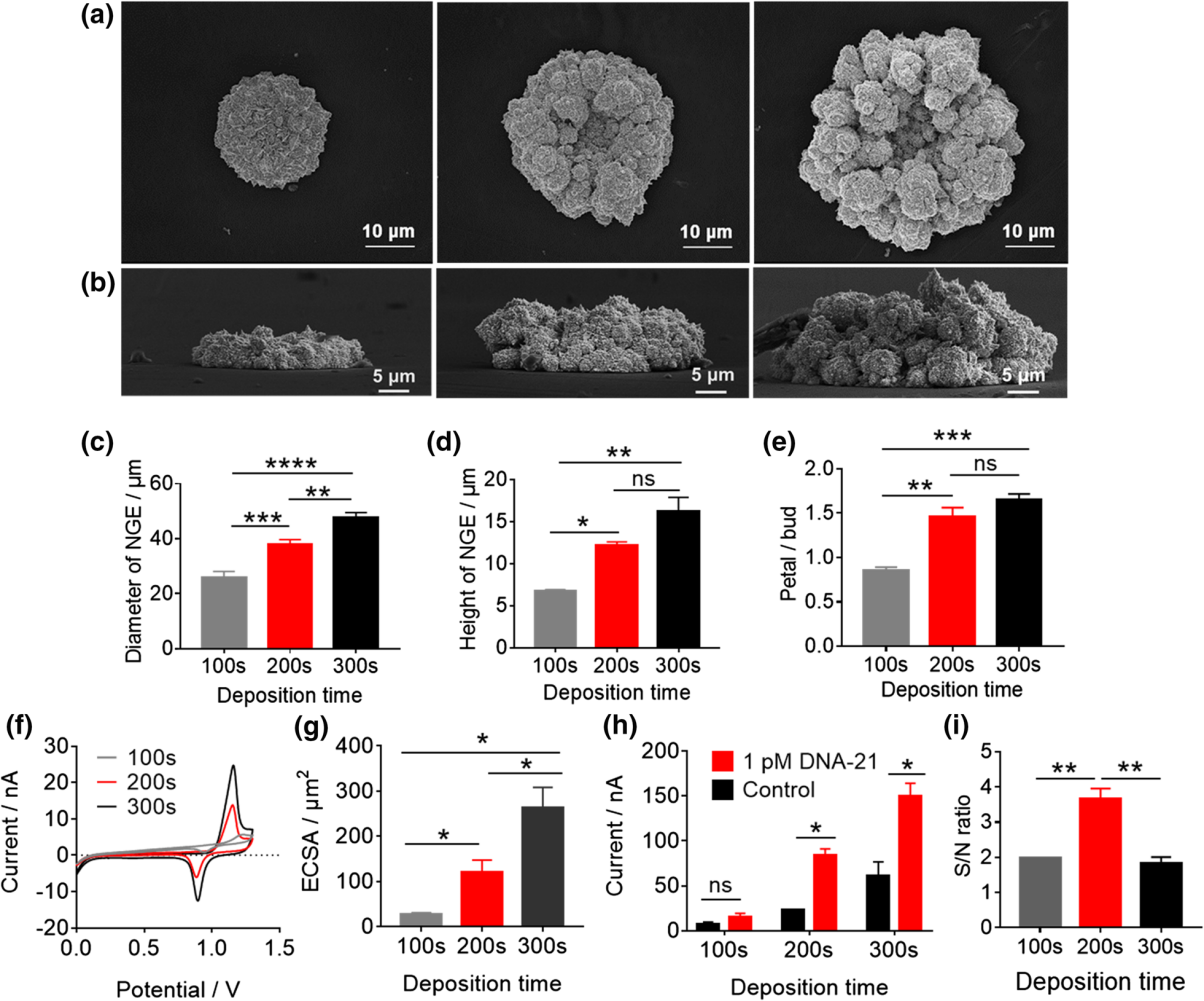
The optimization of electroplating time to fabricate GNE for nucleic acid sensing. The SEM images of the top views (**a**) and side views (**b**) of GNEs with deposition time of 100 s, 200 s and 300 s (left to right). The comparison of diameter (**c**) and height (**d**) of GNEs with different deposition time based on the SEM images of **a** and **b**, respectively. **e** The changes of the “petals” to “bud” ratio of GNEs with the deposition time characterized by SEM imaging. **f** The CV scans of GNEs with different deposition time in 10 mM H_2_SO_4_ with a scan rate of 0.05 V/s. **g** The ECSAs calculated from CVs obtained in **f**. **h** The comparison of the detection performance of 1 pM DNA-21 among the three different sizes of GNEs. The control group was the hybridization buffer without DNA-21. (i) The S/N ratio calculated from **h**. N ≥ 3 per group, ns: not significant, *p < 0.05, **p < 0.01, ***p < 0.001, ****p < 0.0001

It was expected that the size of the GNEs could be a key factor for the molecular sensing performance of the e-miRchip. Thus, we first determined the optimal size of GNEs for miRNA sensing. Three different sizes of GNEs were constructed by controlling the deposition time to be 100 s (GNE_100s_), 200 s (GNE_200s_) and 300 s (GNE_300s_). Despite the different deposition conditions, the grown GNEs all presented a flower shape with “petals” surrounding the central “bud”. As the deposition time increased, both the diameter and the height of GNEs significantly increased (Fig. 3a, b). The average diameter of GNEs increased from 25.86 ± 1.86 nm (GNE_100s_) to 47.81 ± 1.4 nm (GNE_300s_) (Fig. 3c) while the height of GNEs increased from 6.88 ± 0.15 nm to 17.62 ± 1.55 nm, respectively (Fig. 3d). Interestingly, the area ratio of “petal” to “bud” region of GNE_200s_ was higher than that of GNE_100s_, while the ratio was similar between GNE_300s_ and GNE_200s_ (Fig. 3e). This observation suggested that the growth pattern of the GNEs varied depending on the deposition time. Prior to 200 s, the GNE seemed to have a 2-dimensional growth from the edge; after 200 s, a 3-dimensional growth was observed. Such a phenomenon may be explained by the hemispherical diffusion of gold atoms at GNEs, which led to a fast transport at the electrode edge and a slow parallel diffusion at the center of the GNEs [30]. In addition, the ECSA of GNEs were measured by CV scans in H_2_SO_4_ (Fig. 3f). We found the ECSA of GNE increased with the increase of deposition time (Fig. 3g). To choose the optimal deposition time, these GNEs were tested for DNA-21 detection (Fig. 3h). We found that with or without DNA-21 (1 pM), the electrical current increased with the increasing deposition time. Using the ratios of I_DNA-21_ and I_Control_ to represent signal-to-noise (S/N) ratio for the efficient hybridization (Fig. 3i), we found that the GNE_200s_ (with the deposition time of 200 s) had the highest ratio of ~ 3.7, indicating the maximum sensitivity for nucleic acid sensing.

**Fig.4:**
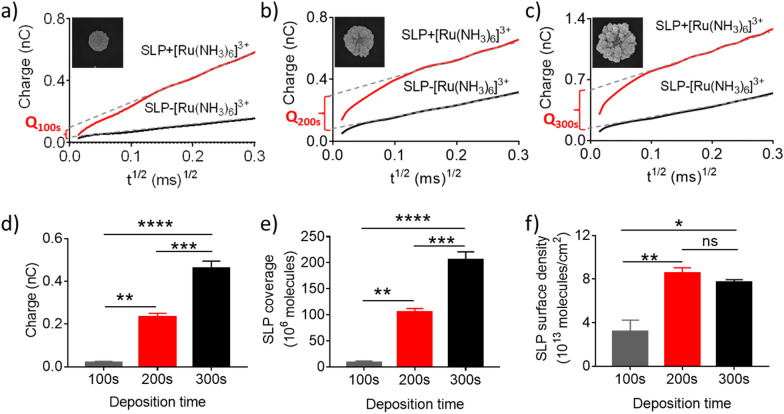
Characterization of the surface charge, SLP coverage and SLP surface density of the GNEs fabricated with different electroplating time. **a**–**c** The chronocoulometry response curves and the SEM images (inset) of the SLP modified GNEs with deposition time of 100 s (**a**), 200 s (**b**), and 300 s (**c**). The calculated surface charges (**d**), SLP coverage (**e**), and the SLP density (**f**) of different GNEs based on the chronocoulometry curves from **a**–**c**. N ≥ 3 per group, ns: not significant, *p < 0.05, **p < 0.01, ***p < 0.001, ****p < 0.0001

In order to understand why GNE_200s_ exhibited a higher signal-to-noise (S/N) ratio compared to GNE_100s_ and GNE_300s_ (Fig. 3i), the chronocoulometry method was applied to calculate the changes of the electrode surface charge introduced by the SLP modification. By analyzing the intercept differences from the two curves measured in the solution with or without [Ru(NH_3_)_6_]^3+^, we were able to calculate the surface coverage and density of SLPs on the three electrodes (GNE_100s_, GNE_200s_, and GNE_300s_) (Fig. 4** a-c**). The results showed that both of the surface charges and the SLP surface coverage followed a trend of GNE_100s_ < GNE_200s_ < GNE_300s_ (Fig. 4d, e). Interestingly, the surface density of SLPs calculated by the Cottrell equation presented a different trend of GNE_100s_ < GNE_200s_ ≈ GNE_300s_ (Fig. 4f). Studies have showed that nanostructured electrodes with a higher probe density usually possess higher hybridization efficiency because of the larger deflection angle of the probes on the electrode surface [44]. Because GNE_100s_ had the lowest surface density of SLPs, it was reasonable that GNE_100s_ displayed a low S/N ratio and hybridization efficiency. For GNE_200s_ and GNE_300s_, although there was no difference in the probe density between them (Fig. 4f), the larger ECSA of GNE_300s_ could increase the nonspecific adsorption of AgNRs, resulting in a higher background current, and hence a lower S/N ratio. Taken together, the 200 s electroplating time appeared to be optimal for GNE fabrication and nucleic acid sensing; accordingly, this condition was used for the following experiments on miR-21 detection.

### Specificity and sensitivity of miRNA detection of the developed e-miRchip

**Fig. 5:**
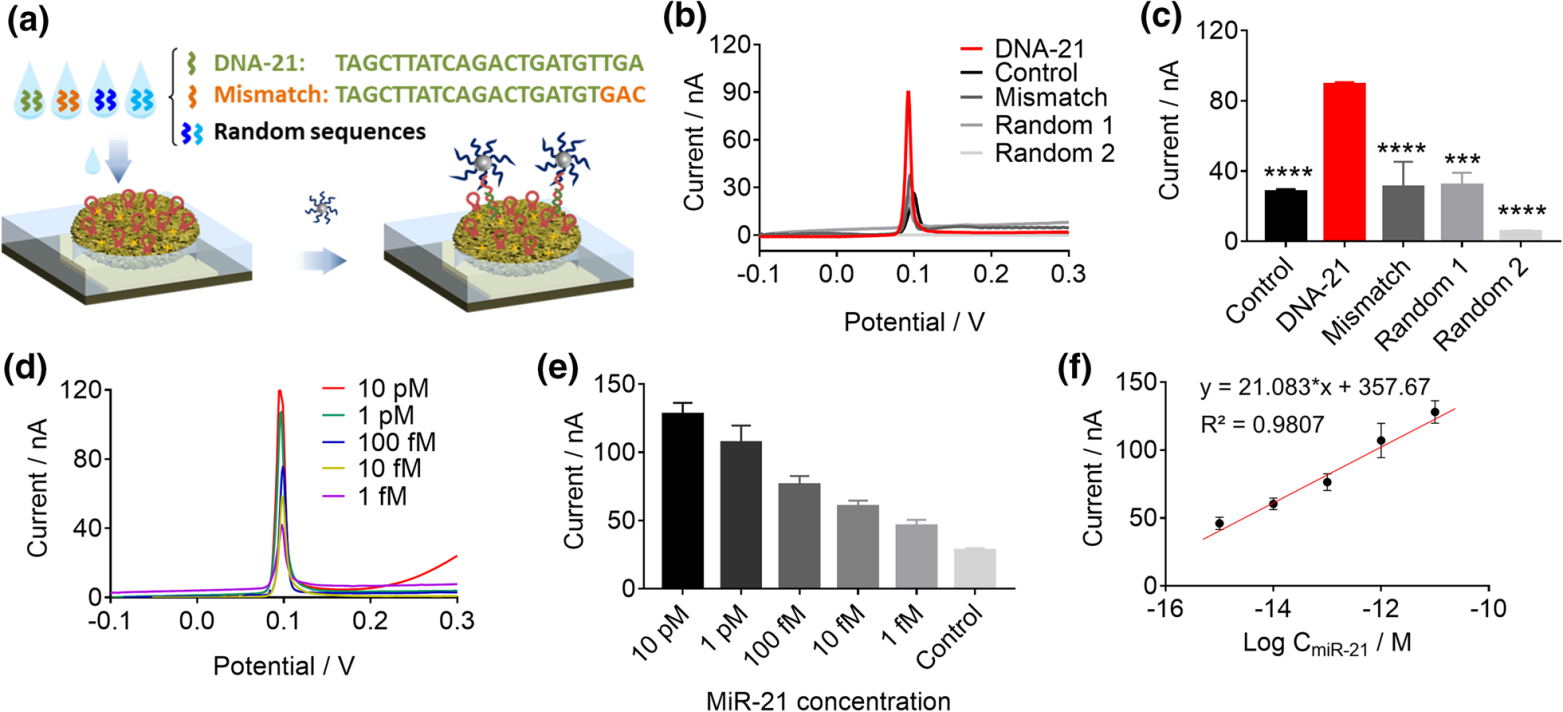
The specificity and sensitivity of e-miRchip in detecting miR-21. **a** A schematic diagram for verification of the specificity of e-miRchip by three non-complementary sequences (a 3-base mismatched sequence and two random sequences) in comparison with the complementary sequence (DNA-21). **b** CVs of the electrooxidation process of e-miRchip in sensing DNA-21, 3 bases mismatched DNA-21 (Mismatch) and two random sequences (Random 1 and Random 2); the concentration of all tested sequences was at 100 fM. **c** The oxidation peak current obtained from **b**. **d** CVs of the electrooxidation process of e-miRchip in the presence of miR-21 at various concentrations from 1 fM to 10 pM. **e** The oxidation peak current obtained from **d**. **f** The calibration curve obtained by plotting the electrooxidation peak current with miR-21 concentrations. N ≥ 3 per group, ***p < 0.001, ****p < 0.0001

Next, we examined the sensing specificity of the e-miRchip (Fig. 5a). Because the stem-loop structure of the SLP can be opened at the stem region of 5’ end when hybridized with the 3’ end of the target, a three-base mismatch sequence at the 3’ end was designed to evaluate the specificity of SLP. By employing this 3-base mismatched sequence and two random sequences in comparison with DNA-21, we found that only the DNA-21 (100 fM) generated the highest signal response (89 nA), while the oxidation current from the two random sequences and the mismatched one were similar to that of the control group (28 nA) (Fig. 5b, c). This indicated that our e-miRchip could distinguish the DNA-21 sequence from the 3-base mismatched one, suggesting an excellent specificity of the e-miRchip for DNA sensing.

For the sensitivity test, the CV curves of the e-miRchip in the presence of various concentrations of miR-21 from 1 fM to 10 pM were obtained (Fig. 5d). The electrooxidation peak current extracted from the CV curves increased with the increase of miR-21 concentrations (Fig. 5e), and a corresponding calibration curve was constructed with a fitting equation of y = 21.083 log C_miR-21_ + 357.67 (R^2^ = 0.9807) (Fig. 5f). From the fitting curve, a limit of detection (LOD) of 0.56 fM was obtained, which was three orders of magnitude lower than that of the macroelectrode (Additional file 1: Fig. S1), indicating the ultrasensitivity of e-miRchip.

### The detection of miR-21 by e-miRchip in heterogeneous biological samples

**Fig. 6:**
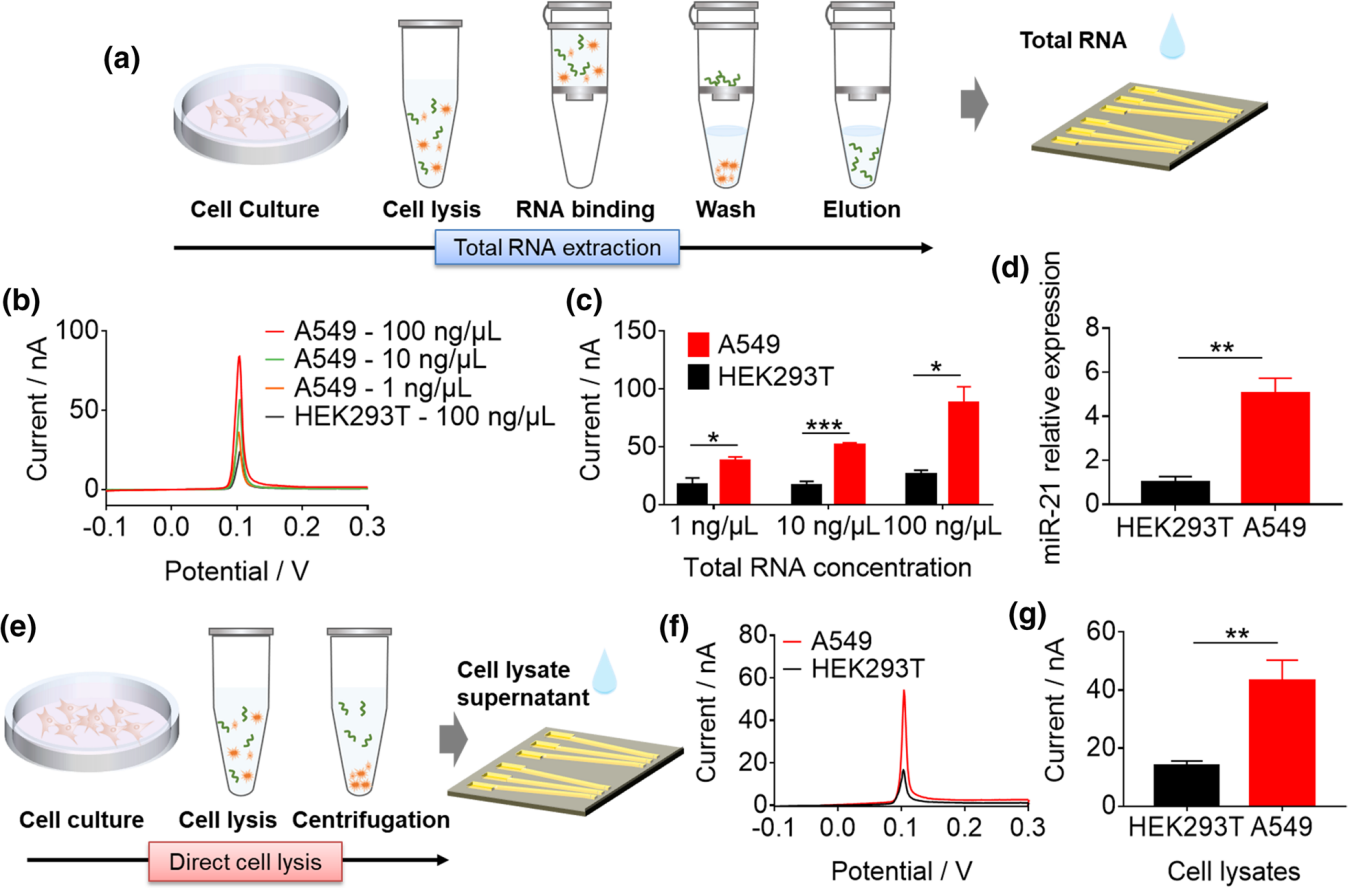
The analysis of miR-21 expression levels by e-miRchip in the total RNA extracts and cell lysates from the A549 lung cancer cells and HEK-293 T cells. **a** The schematic diagram of the workflow for miR-21 detection in total RNA samples obtained from cultured cells. **b** Electrooxidation process of CVs and their peak currents **c** from analyzing the total RNAs of A549 cells at the concentrations of 1 ng/μL, 10 ng/μL and 100 ng/μL, and the total RNA (100 ng/μL) of HEK293T cells. **d** The miR-21 expression in A549 and HEK293T cells measured by RT-qPCR at the total RNA concentration of 2 ng/μL. **e** The schematic diagram of the workflow for the direct detection of miR-21 in cell lysates from A549 and HEK293T cells. **f** Electrooxidation process of CVs and their peak currents **g** for direct miR-21 detection in the cell lysates. N ≥ 3 per group, *p < 0.05, **p < 0.01, ***p < 0.001

To examine the capability of e-miRchip on miRNA detection in complex biological samples, we selected the typical tumor biomarker miR-21 as an example. The purified total RNA samples were extracted from the lung cancer cell line A549. The total RNA extraction was conducted following the procedure shown in Fig. 6a prior to the electrochemical measurement. The performance of e-miRchip in detecting miR-21 was evaluated with the total RNA samples from either A549 or the non-cancer cell HEK293T at three concentrations: 1, 10 and 100 ng/μL. As shown in Fig. 6b, the electrooxidation currents from the A549 samples increased with the increasing concentrations of the total RNA, but that from the HEK293T sample (at 100 ng/μL) remained the lowest. When comparing the electrooxidation peak currents from the A549 samples to those from the HEK293T samples (Fig. 6c), we found that e-miRchip was able to detect miR-21 at the total RNA concentration as low as 1 ng/μL. More importantly, the electrochemical signals remained low in the normal samples regardless the total RNA concentrations, indicating the good performance of e-miRchip in miRNA detection in total RNA samples. To validate our results, RT-qPCR was conducted to analyze the level of miR-21 in the tested samples. As shown in Fig. 6d, the miR-21 expression level in A549 cells was significantly higher than that in HEK293T cells. These results suggested that our developed e-miRchip was able to profile miRNA expression in the total RNA extracts from cells.

To ensure that e-miRchip has the point-of-care testing (POCT) capability, we investigated whether e-miRchip could perform direct detection of miRNAs in the cell lysate without purification. For such a purpose, A549 and HEK293T cells (~ 10^3^ cells) were lysed with lysis buffer containing 1% Triton-X 100, and aliquots of the supernatants of the cell lysate were directly dropped onto the e-miRchip (Fig. 6e). We found that the electrochemical signal from the A549 lysate increased approximately 3 times when compared with that from the HEK293T lysate (Fig. 6f, g), suggesting that e-miRchip had great potential to serve as a POCT device for miRNA profiling in complex biological samples.

It is worth emphasizing that our proposed e-miRchip was simpler in the amplification strategy among all AgNPs-mediated biosensors for miRNA detection (Table 2). The e-miRchip did not require additional amplification system other than AgNPs to achieve comparable or even better detection performance, whereas others utilized effective amplification reactions (e.g., RCA, HCR, etc.) or employed additional nanomaterials (e.g., gold nanoparticles, graphene, etc.) and other biochemical compounds (e.g., biotin/neutravidin, endonuclease, 4-mercaptophenylboronic acid, etc.) to obtain a relative low detection limit. Consequently, the operation step for e-miRchip was greatly simplified and the detection time was reduced. Furthermore, as the developed e-miRchip utilized the microfabrication technology, it was also able to achieve multiplexed, direct and electronic profiling of miRNAs.

**Table 2:** The comparison of different additional amplification strategies for AgNPs-mediated electrochemical miRNA detection

Amplification method	LOD	Linear range	Sample	Refs.
RCA	0.05 fM	0.1 fM–10 nM	Cells/Serum	[24]
HCR	0.39 fM	0.5 fM–1 nM	Cells	[26]
Tetrahedral DNA/SDR	0.4 fM	1 fM–1 nM	Serum	[27]
DSN/AuNPs	0.62 fM	1 fM–1 pM	Plasma	[28]
Walker amplification	32 fM	100 fM–1 μM	Serum	[45]
Graphene/polyaniline	0.2 fM	10 fM–10 μM	Blood	[46]
SDR/polymerase/endonuclease	0.07 fM	1 fM–10 nM	Cells	[47]
AuNPs/biotin/neutravidin	300 fM	500 fM–1 μM	Serum	[48]
SDR/biotin/streptavidin	0.4 fM	1 fM–200 pM	Blood	[49]
Network of MPBA-AgNPs	0.02 fM	0.1 fM–2 pM	Serum	[50]
None	0.56 fM	1 fM–10 pM	Cells	This work

*RCA* rolling circle amplification, *HCR* hybridization chain reaction, *SDR* strand displacement reaction, *DSN* duplex-specific nuclease, *MPBA* 4-mercaptophenylboronic acid

## Conclusion

We developed a simple, ultrasensitive and multiplexed miRNA detection platform, e-miRchip, by combining the electroplated compact gold nanoflower electrode GNE and the signal reporter system AgNRs in a microfabricated electronic array on a glass substrate. The e-miRchip based on the optimal size of GNE showed an ultralow LOD of 0.56 fM (three orders of magnitude lower than that of the macroelectrode) with a wide linearity from 1 fM to 10 pM for miR-21 detection. The e-miRchip exhibited relatively good specificity, where it could easily distinguish 3-base mismatched oligonucleotide sequence from the real target. More importantly, the e-miRchip was able to detect the differential expression of miR-21 in lung cancer cells and in normal cells from total RNA extracts (as low as 1 ng/μL) as well as from the unpurified cell lysates (10^3^ cells). This work demonstrated that the developed e-miRchip could be a new class of highly efficient and promising point-of-care diagnostic devices for the detection of miRNAs, DNA, oligonucleotides and other biological molecules.

## Supplementary Information

**Additional file 1. **The performance of the AgNRs-based miRNA analysis using gold macroelectrode accompanies this paper.

## Data Availability

All data generated and analyzed during this research are included in this article.
