# Supplementary material for: Silver nano-reporter enables simple and ultrasensitive profiling of microRNAs on a nanoflower-like microelectrode array on glass
Source: J Nanobiotechnology. 2022 Oct 23;20:456. doi: 10.1186/s12951-022-01664-7 (PMC9590124; doi:10.1186/s12951-022-01664-7)
Supplement: Supplementary file 1 — Additional file 1. The performance of the AgNRs-based miRNA analysis using gold macroelectrode accompanies this paper. [file 12951_2022_1664_MOESM1_ESM.pdf]

Additional file 1

Silver nano-reporter enables simple and ultrasensitive profiling of microRNAs on a nanoflower-like microelectrode array on glass

Ying Gan [1, a], Mingxing Zhou [2, a], Huiqiang Ma [1], Jiameng Gong [1], Shan-Yu Fung[3], Xian Huang [2,*], Hong Yang [1, *]

[1] The Province and Ministry Co-Sponsored Collaborative Innovation Center for Medical Epigenetics; Department of Pharmacology, School of Basic Medical Sciences; School of Biomedical Engineering; Intensive Care Unit, The Second Hospital, Tianjin Medical University, No. 22 Qixiangtai Road, Heping District, Tianjin 300070, China

[2] Department of Biomedical Engineering, Tianjin University, 92 Weijin Road, Tianjin 300072, China

[3] Department of Immunology, School of Basic Medical Sciences, Tianjin Medical University, No. 22 Qixiangtai Road, Heping District, Tianjin 300070, China

[a] These authors contribute equally to this work

* Corresponding author

Prof. Hong Yang

Email: hongyang@tmu.edu.cn

Prof. Xian Huang

Email: huangxian@tju.edu.cn

The Au macroelectrodes were applied to detect DNA-21 (the corresponding DNA sequence of miR-21) through the proposed two-step hybridization strategy (**Fig. S1a**). The SLPs were first immobilized on the macroelectrode surface through Au-S bond followed by the MCH treatment to block the unbounded sites. In the presence of DNA-21, the hybridization of DNA-21 with SLPs opened the stem-loop structure to expose the 3' terminal sequence, which could subsequently hybridize with the signal probes on the AgNRs. The immobilized AgNRs generated the oxidization and reduction current of Ag during the electrochemical CV scan, and the current signals reflected the amount of the hybridized DNA-21 molecules. Based on this sensing principle, we found that the oxidization peak of AgNRs increased with the increase in the DNA-21 concentration, indicating the positive correlation between the DNA-21 concentrations and AgNRs signals (**Fig. S1b, c**). By plotting the electrooxidation peak current at the various DNA-21 concentrations, a linear relationship between the current and Log C_{target} was obtained, with a fitting equation of y = 18.54 Log C_{target} + 116.7 (R^2 = 0.9988), where the limit of detection (LOD) was calculated to be 0.71 pM (**Fig. S1d**).

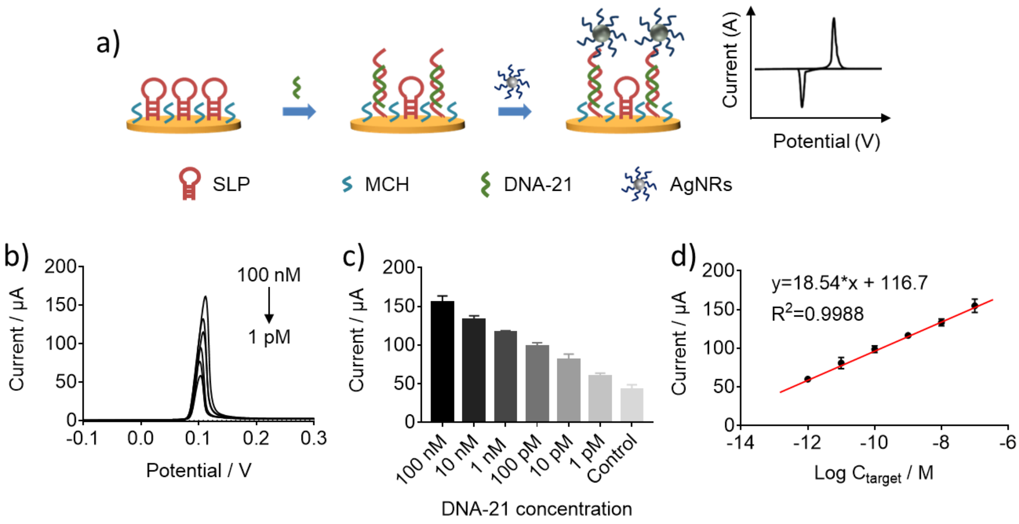

Fig. S1 Detection of DNA-21 using AgNRs mediated two-step hybridization strategy on an Au macroelectrode. (a) Schematic diagram of the macroelectrode sensing platform using the two-step hybridization strategy to detect DNA-21 (the corresponding DNA sequence of miR-21). (b) The representative CV curves for the detection of DNA-21 at various concentrations from 1 pM to 100 nM. (c) Extracted electrooxidation peak current from the CV curves in (b). (d) The fitting curve obtained by plotting the electrooxidation peak current of AgNRs at various concentrations of DNA-21 (Log C_{target}). N=3 per group.
